# Supplementary material for: A consistent map in the medial entorhinal cortex supports spatial memory
Source: Nat Commun. 2024 Feb 17;15:1457. doi: 10.1038/s41467-024-45853-4 (PMC10874432; doi:10.1038/s41467-024-45853-4)
Supplement: Supplementary file 1 — Supplementary Information [file 41467_2024_45853_MOESM1_ESM.pdf]

Supplementary Information

A consistent map in the medial entorhinal cortex supports spatial memory

Taylor J. Malone[1,7], Nai-Wen Tien[1,5,7], Yan Ma[1,7], Lian Cui[1], Shangru Lyu[1], Garret Wang[1], Duc Nguyen[1,6], Kai Zhang[1,2], Maxym V. Myroshnychenko[3], Jean Tyan[1], Joshua A. Gordon[3,4], David A. Kupferschmidt[3], Yi Gu[1,*]

[1]Spatial Navigation and Memory Unit, National Institute of Neurological Disorders and Stroke, National Institutes of Health, Bethesda, MD 20892, USA
[2]Department of Anesthesiology, Tianjin Medical University General Hospital, Tianjin, China
[3]Integrative Neuroscience Section, National Institute of Mental Health, National Institutes of Health, Bethesda, MD 20892, USA
[4]Office of the Director, National Institute of Mental Health, National Institutes of Health, Bethesda, MD 20892, USA
[5]Current address: Washington University School of Medicine in St. Louis, St. Louis, MO, USA
[6]Current address: Center of Neural Science, New York University, New York, NY, USA
[7]These authors contributed equally to this work
*Corresponding author: yi.gu@nih.gov

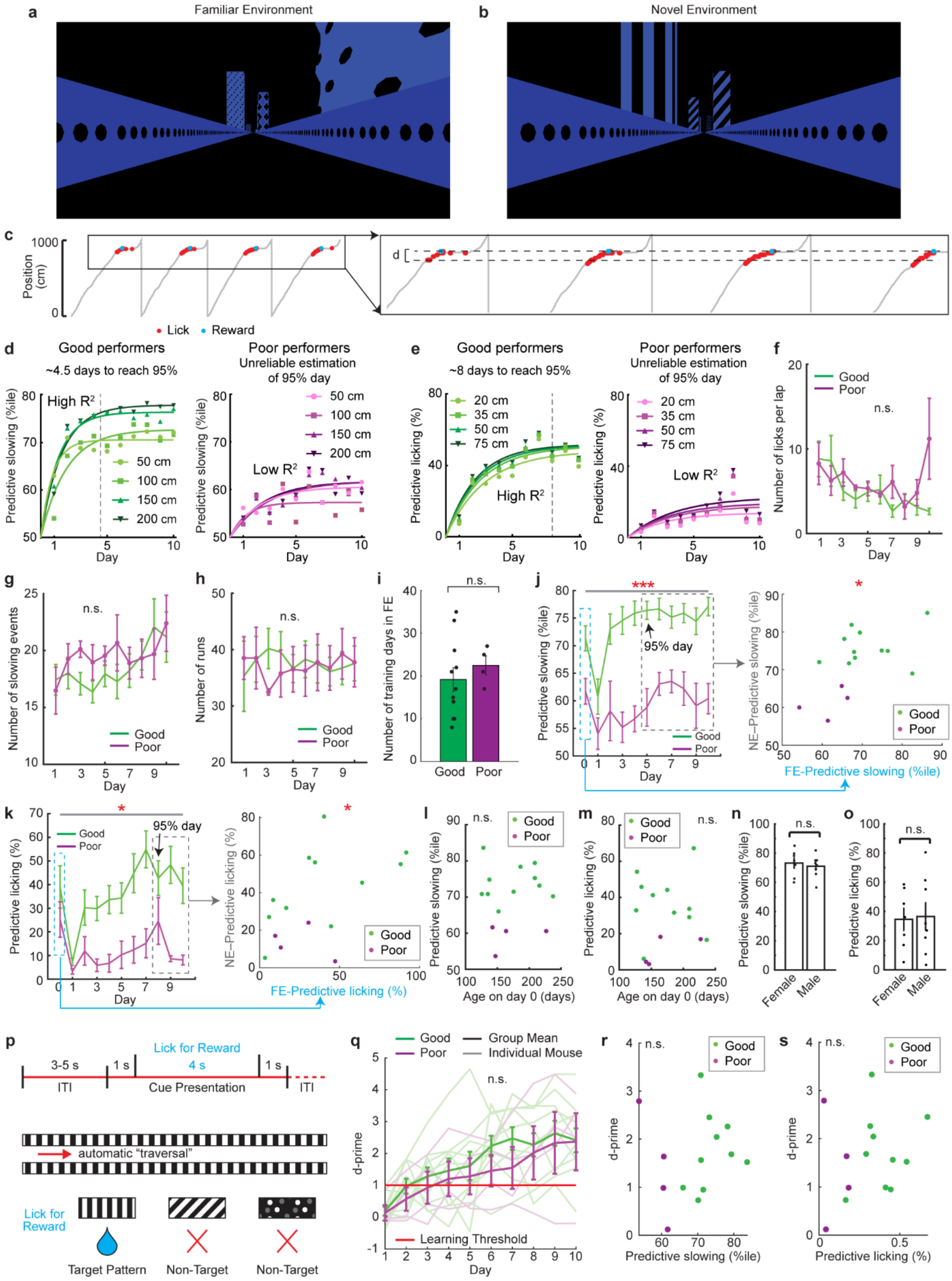
a
Familiar Environment
b
Novel Environment
c
1000
Position (cm)
0
d
Lick
Reward
d
Good performers
~4.5 days to reach 95%
High R²
50 cm
100 cm
150 cm
200 cm
Predictive slowing (%ile)
Day
Poor performers
Unreliable estimation of 95% day
Low R²
e
Good performers
~8 days to reach 95%
20 cm
35 cm
50 cm
75 cm
High R²
Predictive licking (%)
Poor performers
Unreliable estimation of 95% day
Low R²
f
Good
Poor
n.s.
Number of licks per lap
g
n.s.
Number of slowing events
h
n.s.
Number of runs
i
n.s.
Number of training days in FE
Good
Poor
j
***
95% day
NE–Predictive slowing (%ile)
FE-Predictive slowing (%ile)
*
k
*
95% day
Predictive licking (%)
NE–Predictive licking (%)
FE-Predictive licking (%)
l
n.s.
Age on day 0 (days)
m
n.s.
n
n.s.
Female
Male
o
n.s.
p
Lick for Reward
3-5 s
1 s
4 s
1 s
ITI
Cue Presentation
ITI
automatic "traversal"
Lick for Reward
Target Pattern
Non-Target
Non-Target
q
Group Mean
Individual Mouse
n.s.
d-prime
Learning Threshold
r
n.s.
Predictive slowing (%ile)
s
n.s.
Predictive licking (%)

Supplementary Figure 1. Behavior of good and poor performers in virtual reality, Related to Figure 2

a and **b**. Example views from a mouse's perspective of the familiar (FE) (**a**) and novel (NE) (**b**) linear track environment at 300 cm along the track. **c**. Example of the distance threshold (d) used to determine predictive slowing and licking. **d** and **e**. Average days to reach 95% of the plateau for predictive slowing (**d**) and predictive licking (**e**) under different distance thresholds (For the good performers: 4.5 ± 0.6 days for PS and 8 ± 0.4 days for PL. No valid days were determined for the poor performers due to low R^2 values and thus the models cannot reflect the learning stages in poor performers.). R^2 values: for **d** (50cm, 100cm, 150cm, 200cm), good performers (0.63, 0.82, 0.88, 0.90), poor performers (0.43, 0.13, 0.59, 0.49); for **e** (20cm, 35cm, 50cm, 75cm), good performers (0.80, 0.83, 0.85, 0.83), poor performers (0.26, 0.21, 0.23, 0.30).

From **f** to **o**:

Here we demonstrate that PS and PL reflected a mouse's spatial learning ability, rather than other factors, such as attention, experience, environmental features, age, or sex. First, while behavioral disengagement and lack of attention have been shown to affect spatial learning performance of mice[1,2], both good and poor performers exhibited comparable levels of licking and slowing during our task (**Fig. S1f, S1g**), indicating that both groups were engaged to pursue water rewards. Second, the two groups experienced comparable numbers of runs in the NE and training days in the FE, excluding lack of experience as the source of the difference between groups (**Fig. S1h, S1i**). Finally, learning performance was independent of environmental features, mouse age, and sex (**Fig. S1j-o**).

f-h. Number of licks per lap (**f**, excluding licks within 30cm after reward delivery), slowing events (**g**), and runs (**h**) in the novel environment (NE). **i**. Number of training days in the FE. **j** and **k**. Correlation between PS (**j**) and PL (**k**) in FE (on day 0) and NE (after good performers reach 95% of plateau as in **d** and **e**). **l** and **m**. Correlation between PS (**l**) and PL (**m**) and mouse ages on day 0. **n** and **o**. PS (**n**) and PL (**o**) for male and female mice. **p**. Visual discrimination task schematic. ITI: inter-trial interval. **q**. Discrimination factor (d-prime) for the visual discrimination task. **r** and **s**. Correlation between PS (**r**) or PL (**s**) averaged across 10 days in the NE and d-prime averaged across 10 days of the visual discrimination task. Only 14 mice (10 good, 4 poor) are included due to the death of one mouse. *$p \leq 0.05$, **$p \leq 0.01$, ***$p \leq 0.001$. Error bars represent mean ± sem. See Supplementary Data 1 for exact n and detailed statistical information. Source data are provided as a Source Data file.

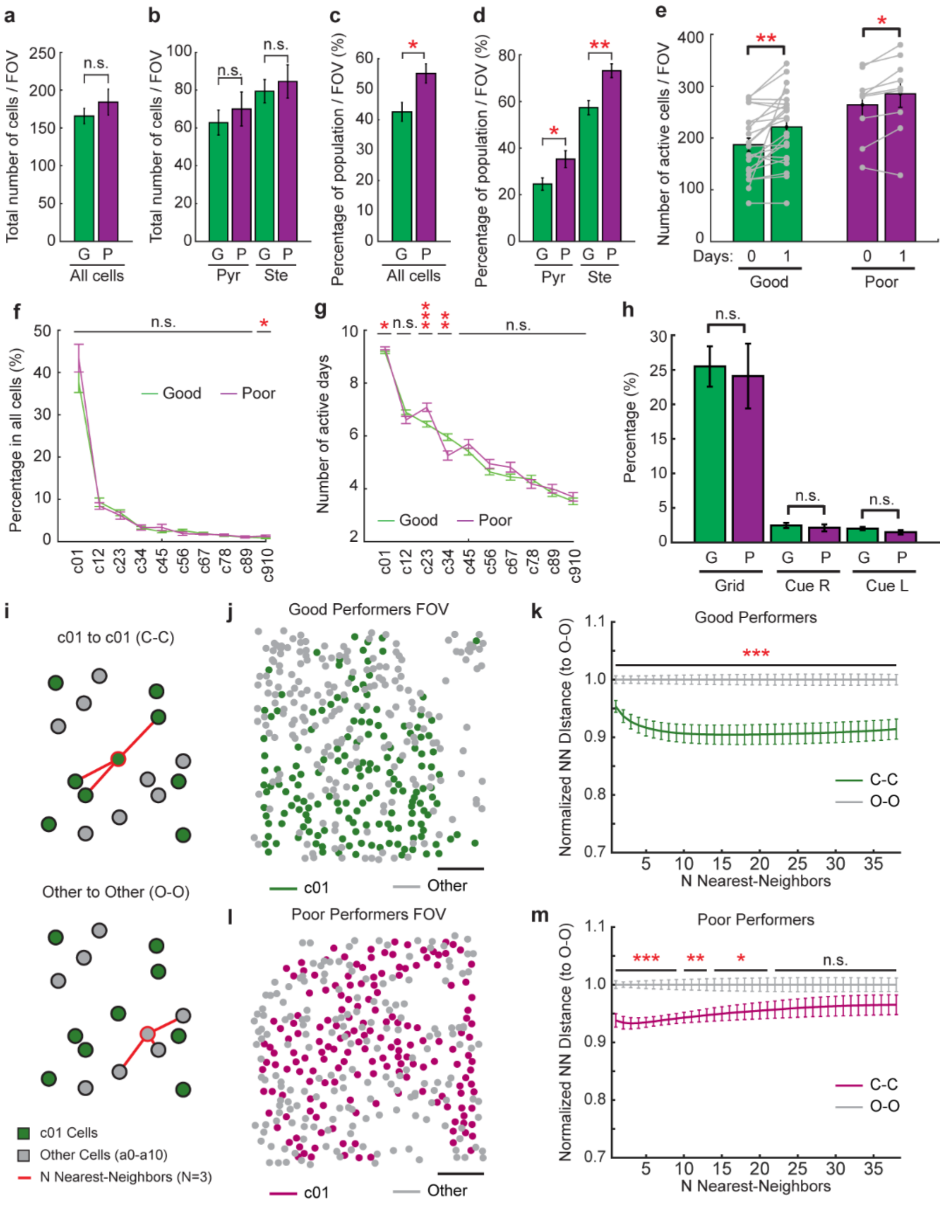

n

Source	p value
Performance (Good vs. Poor)	0.2456
Cluster Size (N)	0.2247
Cluster Type (C-C vs. O-O)	< 0.0001 ***
Performance x Cluster Size	< 0.0001 ***
Performance x Cluster Size x Cluster Type	< 0.0001 ***

Supplementary Figure 2. Properties of the active neural ensemble of the MEC during spatial learning, Related to Figure 2

a and **b**. Number of manually identified cells (**a**) and pyramidal (Pyr) and stellate (Ste) cells (**b**) per FOV. G: good performers; P: poor performers. **c** and **d**. Percentage of manually identified cells (**c**) and Pyr and Ste cells (**d**) per FOV that are active. **e**. Number of active cells per FOV on days 0 and 1. **f**. Percentage of cells newly active on different adjacent pairs of days among the total cell

population. **g**. Number of active days of the cell populations in panel **f**. **h**. Percentage of identified grid, right cue (Cue R), and left cue (Cue L) cells among all cells from all categories in **Fig. 2k**. **i**. Nearest-neighbor anatomical clustering method measuring c01 to c01 (C-C, top) and other cell to other cell (O-O, bottom) distances. **j** and **l**. Example FOV showing centroid positions of c01cells (green/magenta) and other cells (a0-a10, gray) for good (**j**) and poor (**l**) performers. Scale bar: 100μm. **k** and **m**. Nearest-neighbor distances normalized to the mean O-O value for a given N nearest-neighbors in good (**k**) and poor (**m**) performers. **n**. Two-way ANOVA statistics showing a significant interaction between performer group and cluster size. Calculated using all cluster sizes (N nearest-neighbors) between 1 and 38. *$p \leq 0.05$, **$p \leq 0.01$, ***$p \leq 0.001$. Error bars represent mean ± sem. See Supplementary Data 1 for exact n and detailed statistical information. Source data are provided as a Source Data file.

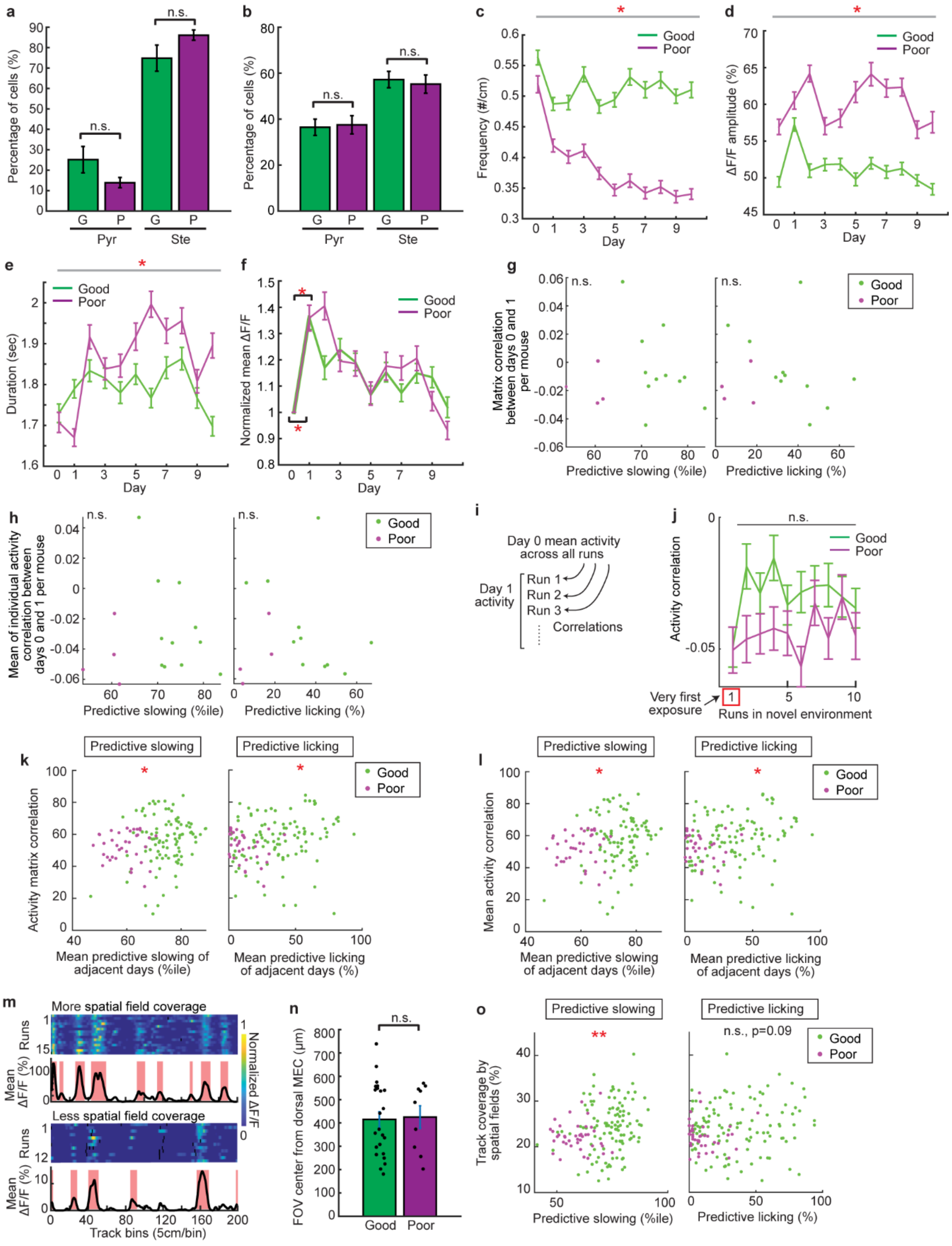
a
Percentage of cells (%)
n.s.
G
P
Pyr
Ste
b
c
Frequency (#/cm)
Good
Poor
Day
d
ΔF/F amplitude (%)
e
Duration (sec)
f
Normalized mean ΔF/F
g
Matrix correlation between days 0 and 1 per mouse
Predictive slowing (%ile)
Predictive licking (%)
h
Mean of individual activity correlation between days 0 and 1 per mouse
i
Day 0 mean activity across all runs
Day 1 activity
Run 1
Run 2
Run 3
Correlations
j
Activity correlation
Very first exposure
Runs in novel environment
k
Predictive slowing
Predictive licking
Activity matrix correlation
Mean predictive slowing of adjacent days (%ile)
Mean predictive licking of adjacent days (%)
l
Mean activity correlation
m
More spatial field coverage
Less spatial field coverage
Runs
Mean ΔF/F (%)
Normalized ΔF/F
Track bins (5cm/bin)
n
FOV center from dorsal MEC (μm)
Good
Poor
o
Track coverage by spatial fields (%)
n.s., p=0.09

Supplementary Figure 3. Neural activity of good and poor performers during learning, Related to Figure 3

a. Percentages of pyramidal (Pyr) and stellate (Ste) cells in the cell population that were persistently active for 11 days. G: good performers. P: poor performers. **b**. Percentages of Pyr and Ste cells in the whole active cell population. **c-e**. Frequency (**c**, represented by the number of significant transients per cm.), calcium response amplitude (**d**), and duration (**e**, seconds) of significant calcium transients. **f**. Mean $\Delta F/F$ normalized to day 0 activity by cell. * indicates the statistical difference between the normalized mean $\Delta F/F$ on days 0 and 1. **g** and **h**. The correlation between the matrix correlation (**g**) or mean activity correlation of individual cells (**h**) on days 0 and 1 (remapping) and predictive slowing (left) and licking (right) on an individual-mouse basis. **i**. A diagram showing the calculation of remapping on a run-by-run basis using the first 10 runs in the first FOV imaged on day 1 in the NE. 10 is the minimal number of runs included in the data of the first FOV across all 15 mice. **j**. The correlation between the activity in the FE and that on the first 10 runs in the NE. **k** and **l**. The correlation between the matrix correlation (**k**) or mean activity correlation of individual cells (**l**) on adjacent days and mean predictive slowing (left) or licking (right) on adjacent days of individual mice. **m**. Examples of neural calcium responses of two cells with more (top) or less (bottom) spatial field coverage. For each cell: top: run-by-run calcium response; bottom: mean $\Delta F/F$. Spatial fields are indicated in red. **n**. FOV center locations from the dorsal border of the MEC. **o**. Correlation between the percentage of track covered by spatial fields and predictive slowing (left) or licking (right). *$p \leq 0.05$, **$p \leq 0.01$, ***$p \leq 0.001$. Error bars represent mean ± sem. Horizontal grey bars indicate p values for the group difference between good and poor performers. See Supplementary Data 1 for exact n and detailed statistical information. Source data are provided as a Source Data file.

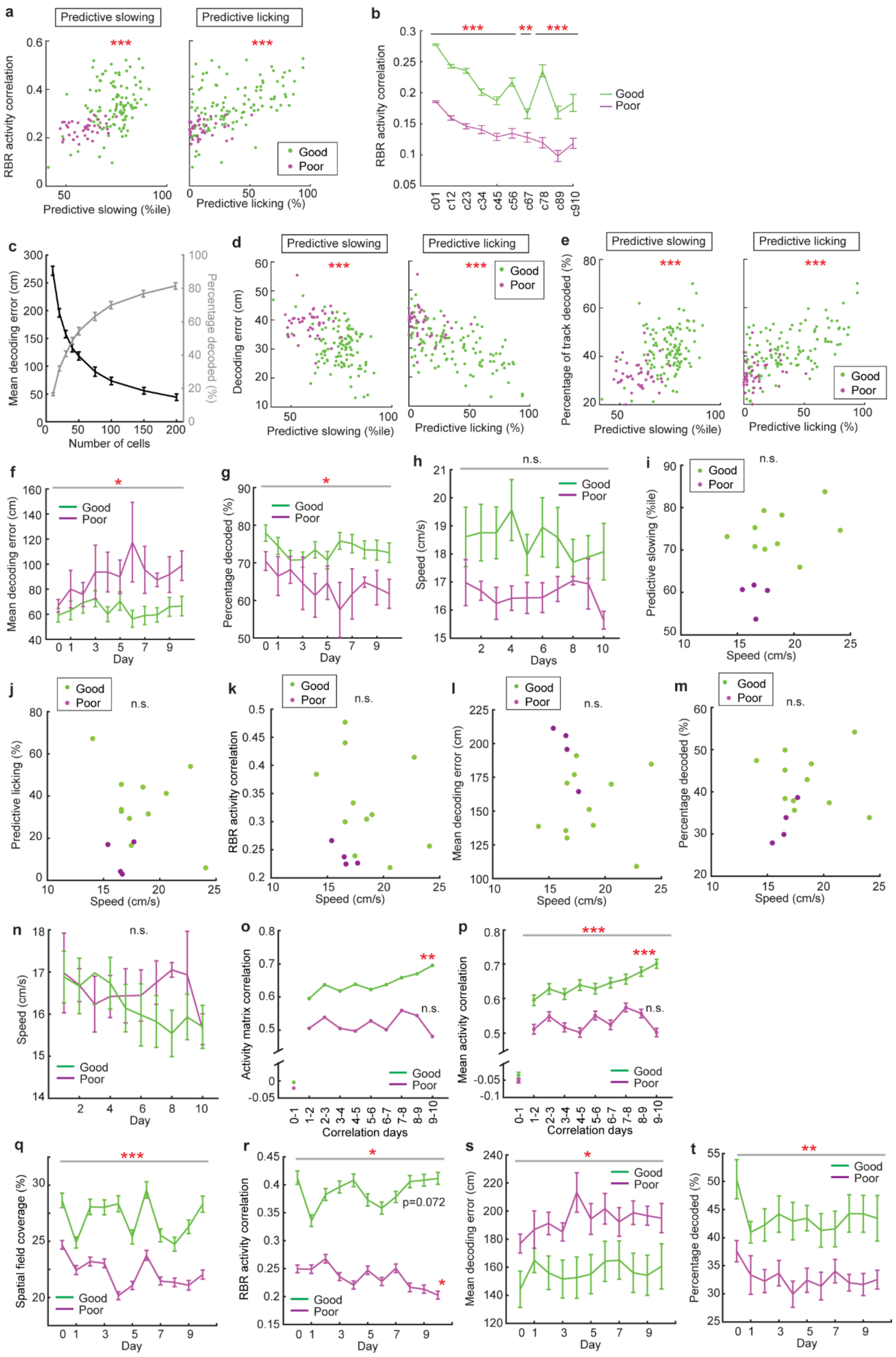
a
Predictive slowing
Predictive licking
***
RBR activity correlation
Predictive slowing (%ile)
Predictive licking (%)
Good
Poor
b
*** ** ***
RBR activity correlation
c01 c12 c23 c34 c45 c56 c67 c78 c89 c910
Good
Poor
c
Mean decoding error (cm)
Percentage decoded (%)
Number of cells
d
Predictive slowing
Predictive licking
Decoding error (cm)
Predictive slowing (%ile)
Predictive licking (%)
e
Predictive slowing
Predictive licking
Percentage of track decoded (%)
f
Mean decoding error (cm)
Day
g
Percentage decoded (%)
Day
h
n.s.
Speed (cm/s)
Days
i
n.s.
Predictive slowing (%ile)
Speed (cm/s)
j
n.s.
Predictive licking (%)
Speed (cm/s)
k
n.s.
RBR activity correlation
Speed (cm/s)
l
n.s.
Mean decoding error (cm)
Speed (cm/s)
m
n.s.
Percentage decoded (%)
Speed (cm/s)
n
n.s.
Speed (cm/s)
Day
o
Activity matrix correlation
Correlation days
0-1 1-2 2-3 3-4 4-5 5-6 6-7 7-8 8-9 9-10
p
Mean activity correlation
Correlation days
q
Spatial field coverage (%)
Day
r
RBR activity correlation
p=0.072
Day
s
Mean decoding error (cm)
Day
t
Percentage decoded (%)
Day

Supplementary Figure 4. Relationship between reward-predictive behaviors, intra-day activity, spatial coding, and speed, Related to Figure 4

a. Correlation between run-by-run (RBR) activity correlation and predictive slowing (left) or licking (right) on individual days of individual mice. **b**. RBR activity correlation of cell populations newly active on different adjacent pairs of days, as categorized in **Fig. S2f**. The correlation for each cell was the mean run-by-run activity correlation of all its active days. **c**. Changes in decoding performance as a function of number of cells used in the decoding analysis. **d**. Correlation between mean decoding error (**d**) or percentage of correctly decoded track positions (**e**) and predictive slowing (left) or licking (right) on individual days of individual mice. **f** and **g**. Mean decoding error (**e**) and percentage of correctly decoded track positions (**f**) using all cells.

From **h** to **t**:

It was previously reported that higher running speed leads to more precise spatial encoding by the MEC[3]. In our study, although the good performers exhibited a trend of higher mean running speeds than the poor performers ($p>0.05$, **Fig. S4h**), the running speed of individual mice was uncorrelated to their PS, or PL (**Fig. S4i, S4j**). Mean speed was also uncorrelated to activity features, such as RBR activity correlation and decoding accuracy (**Fig. S4k-S4m**). To confirm that the activity differences between the good and poor performers were not caused by running speed differences, we excluded six good performers, the running speeds of which on at least one day exceeded the maximal running speed of poor performers. While the remaining five good performers exhibited comparable running speeds to poor performers (**Fig. S4n**), their activity features compared to the poor performers remained consistent with our previous results, including the higher inter-day activity correlation, spatial field coverage, RBR activity correlation, and decoding accuracy (**Fig. S4o-t**). These results demonstrate that the activity differences between the good and poor performers were not due to differences in running speed.

h. Running speeds. **i-m**. Correlations between running speed and predictive slowing (**i**), predictive licking (**j**), RBR activity correlation (**k**), decoding error (**l**), and percentage of correctly decoded track positions (**m**). **n-t**. Comparisons between good and poor performers after removing the six good performers that ran faster than poor performers. Running speed (**n**), matrix correlation (**o**) or mean activity correlation of individual cells (**p**) on adjacent days, percentage of track covered by spatial fields (**q**), RBR activity correlation (**r**), and mean decoding error (**s**) or percentage of correctly decoded track positions (**t**) using 50 randomly selected cells. **s** and **t**. For each FOV, two random selections of 50 cells were made and the results were averaged. $*p \leq 0.05$, $**p \leq 0.01$, $***p \leq 0.001$. Error bars represent mean ± sem. Horizontal grey bars indicate p values for the group difference between good and poor performers. * to the right of line graph indicates significant positive or negative correlation from day 1 to day 10. See Supplementary Data 1 for exact n and detailed statistical information. Source data are provided as a Source Data file.

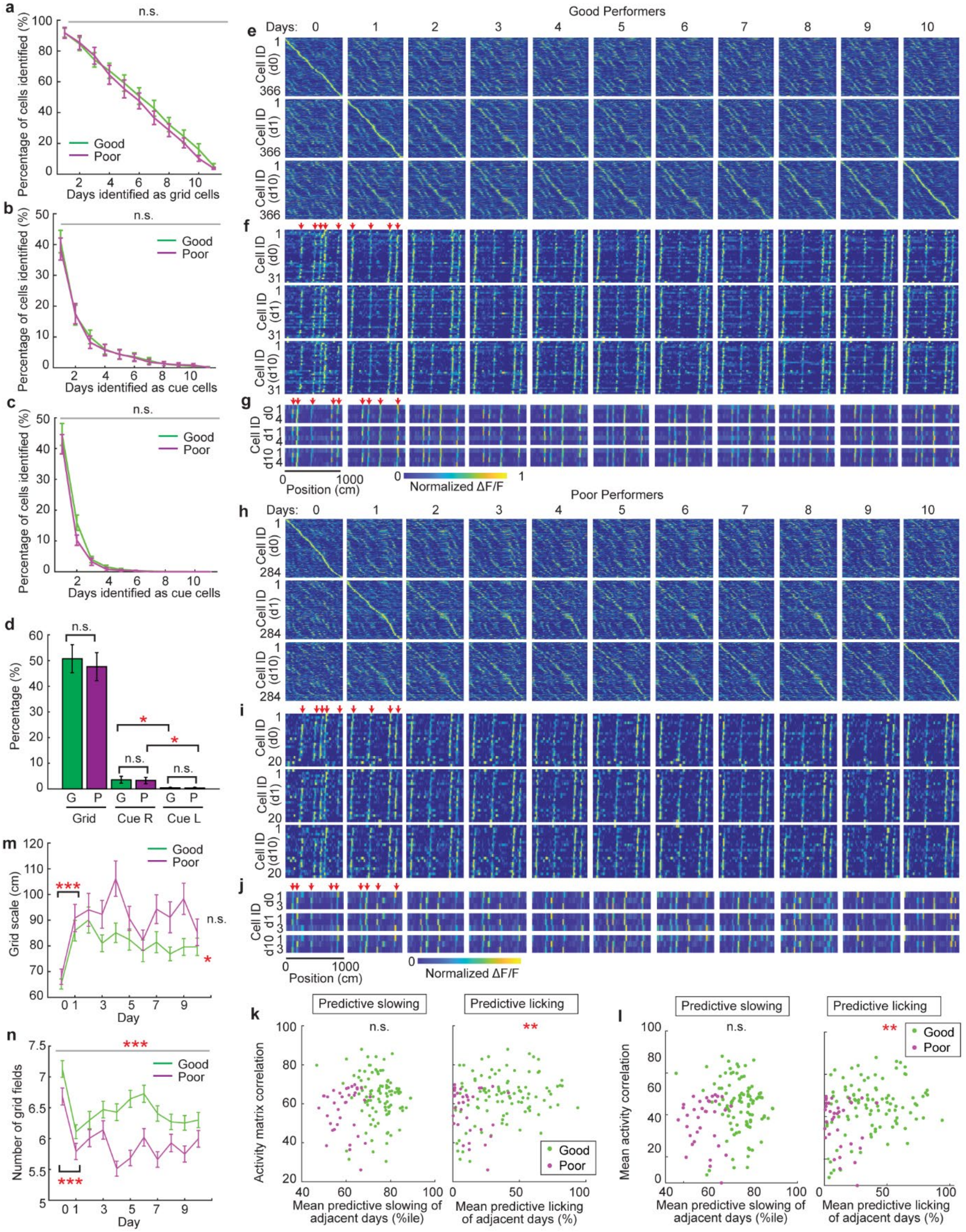
a
b
c
d
e
f
g
h
i
j
k
l
m
n
n.s.
Percentage of cells identified (%)
Days identified as grid cells
Days identified as cue cells
Good
Poor
Percentage (%)
Grid
Cue R
Cue L
G
P
Grid scale (cm)
Number of grid fields
Day
Good Performers
Poor Performers
Days:
Cell ID
Position (cm)
Normalized ΔF/F
Predictive slowing
Predictive licking
Activity matrix correlation
Mean activity correlation
Mean predictive slowing of adjacent days (%ile)
Mean predictive licking of adjacent days (%)

Supplementary Figure 5. Inter-day consistency of neural dynamics of grid and cue cells during spatial learning, Related to Figure 5

a-c. Percentages of grid (**a**), right cue (**b**), and left cue (**c**) cells identified for different numbers of days during the 11 days of imaging among all persistent cells. **d**. Percentages of grid (Grid), right cue (Cue R), and left cue (Cue L) cells identified as those cell types on at least 6 days among all persistent cells. **e-j**. Activity matrices of grid (**e**, **h**), right cue (**f**, **i**), and left cue (**g**, **j**) cells for good (**e-g**) and poor (**h-j**) performers. The normalized mean ΔF/F of individual cells were sorted based on the peak locations on day 0. **f**, **g**, **i**, **j**. The right and left cue locations in familiar and novel environments are indicated by the red arrows. **k** and **l**. The correlation between the matrix correlation (**k**) or mean activity correlation of individual cells (**l**) on adjacent days for grid cells and mean predictive slowing (left) or licking (right) on adjacent days of individual mice. **m** and **n**. Change in grid scales (**m**) and the number of grid fields (**n**) during learning. The * between days 0 and 1 indicates a significant difference for both good and poor performers. * to the right of line graph indicates significant correlation from day 1 to day 10. *$p \leq 0.05$, **$p \leq 0.01$, ***$p \leq 0.001$. Error bars represent mean ± sem. Horizontal grey bars indicate p values for the group difference between good and poor performers. See Supplementary Data 1 for exact n and detailed statistical information. Source data are provided as a Source Data file.

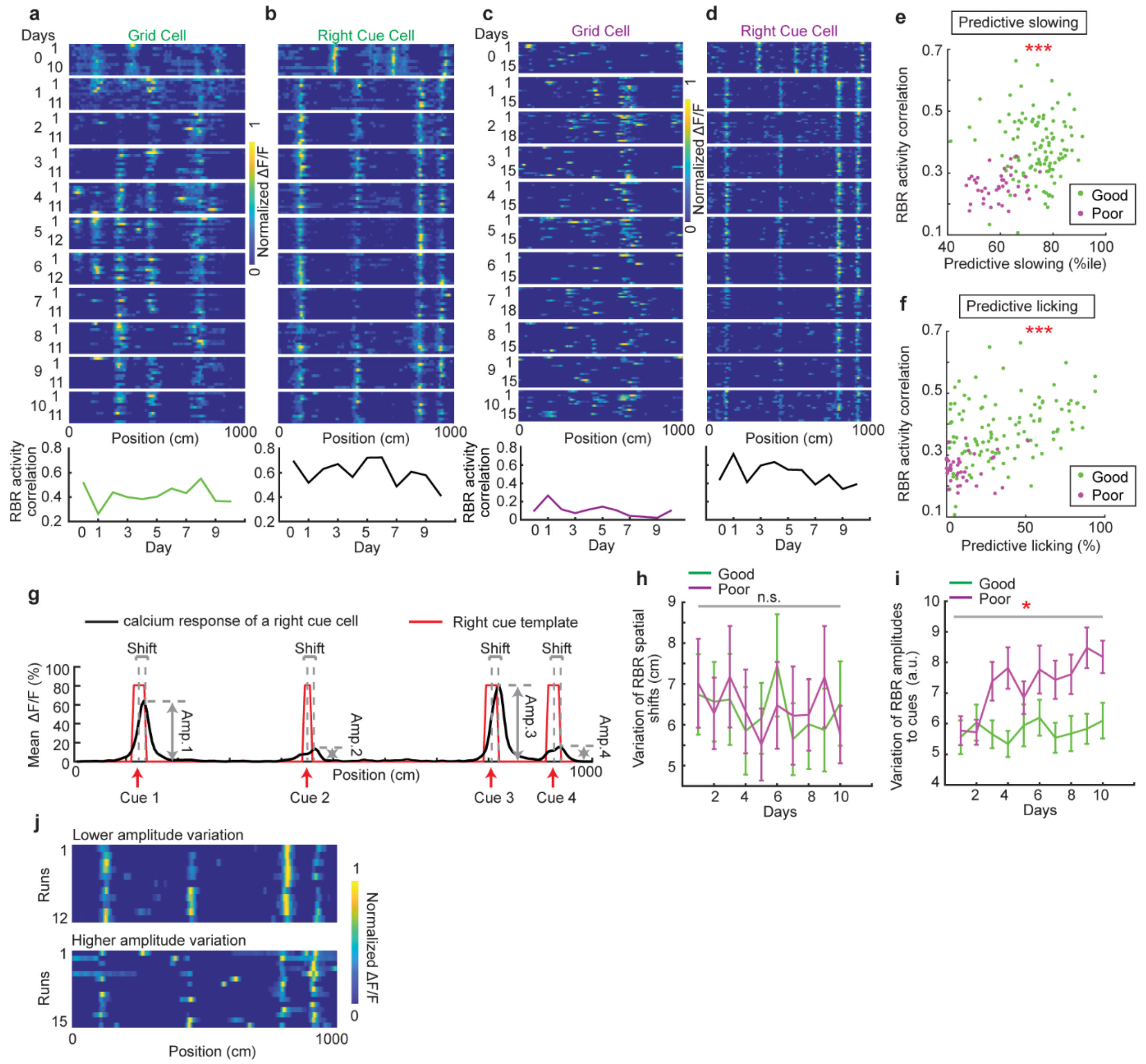

Supplementary Figure 6. Intra-day consistency of grid and cue cells during spatial learning, Related to Figure 5

a-d. Panels 1 to 11 (from top to bottom): Example run-by-run (RBR) calcium response of a simultaneously imaged pair of a grid (**a**, **c**) and a right cue (**b**, **d**) cell from days 0 to 10 from a good (**a**, **b**) or poor (**c**, **d**) performer. Bottom Panel: RBR activity correlation of example cell. **e** and **f**. Correlation between the RBR activity correlations of grid cells and predictive slowing (**e**) or licking (**f**) on individual days of individual mice. **g**. Activity features of a right cue cell, which shows consistent spatial response shifts but different response amplitudes to individual cues. **h** and **i**. Variation (standard deviation) of spatial activity shifts (**h**) and response amplitudes (**i**) on an RBR basis during the 10 days in the novel environment. The data of both right and left cue cells were included. **j**. Cue cell examples for good (top) and poor (bottom) performers with largely consistent spatial shifts but high (good) and low (poor) response amplitude variations to individual cues. *p $\leq$ 0.05, **p $\leq$ 0.01, ***p $\leq$0.001. Error bars represent mean ± sem. Horizontal grey bars

indicate p values for the group difference between good and poor performers. See Supplementary Data 1 for exact n and detailed statistical information. Source data are provided as a Source Data file.

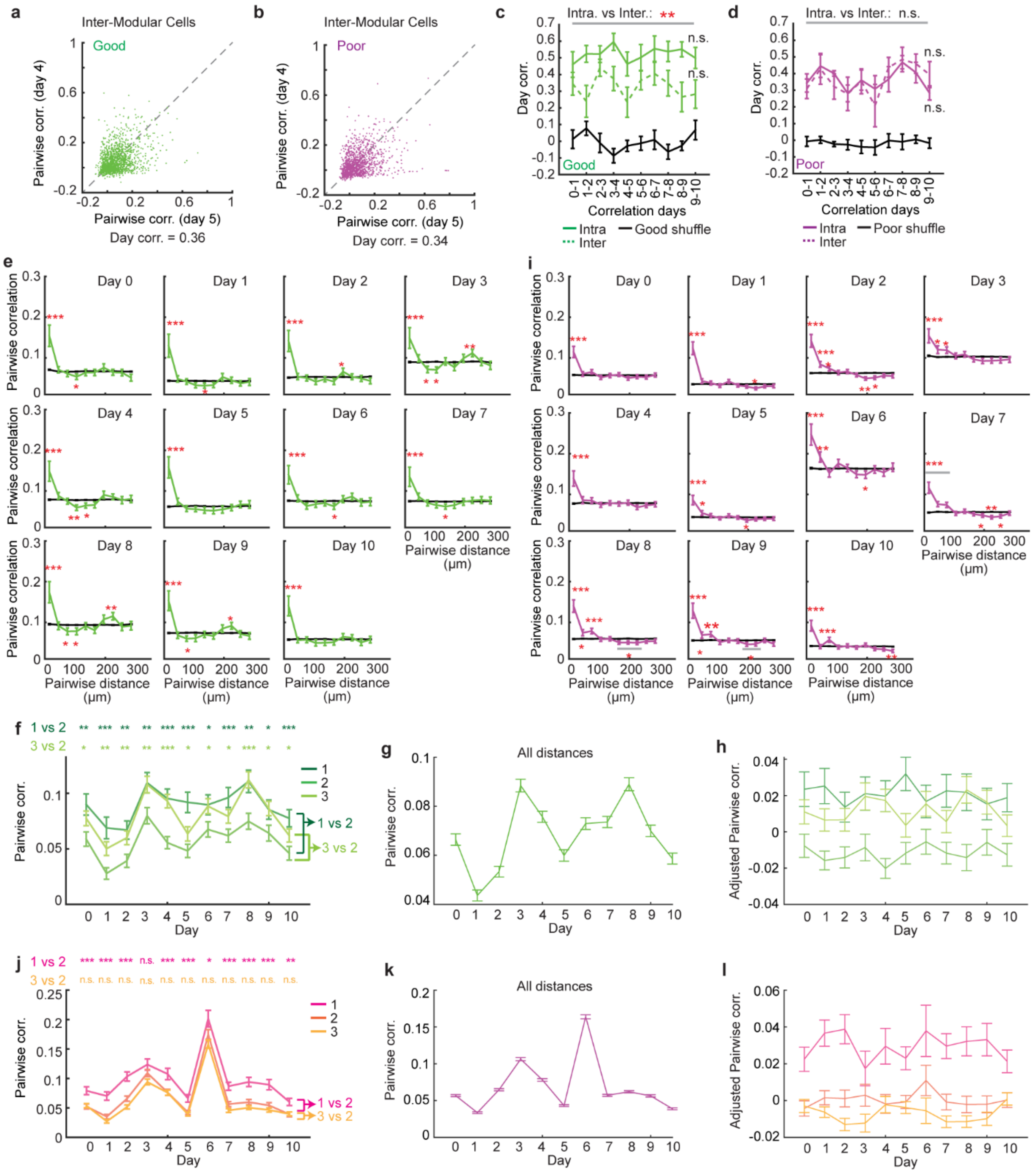

Supplementary Figure 7. Pairwise grid activity correlations as a function of anatomical distance during learning, Related to Figure 5

a and **b**. Pairwise activity correlations (corr.) of grid cells in different modules on days 4 and 5 in good (**a**) and poor (**b**) performers. Day corr. indicates the correlation between Pairwise corr. on the two days. **c**. and **d**. Day corr. for intra-modular cells, inter-modular cells, and shuffles in good (**c**) and poor performers (**d**). Error bars are generated by data from individual FOVs. Horizontal grey bar indicates the group difference between intra-modular correlation compared to inter-

modular correlation. n.s. to the right of the line graph indicates no significant correlation across day pairs. **e** and **i**. Pairwise activity correlations as a function of pairwise anatomical distance for good (**e**) and poor (**i**) performers on individual days, calculated using the ΔF/F constructed by significant calcium transients. * indicates the significant difference between real data and shuffles. **f** and **j**. Pairwise corr. In the three zones in good (**f**) and poor (**j**) performers. **g** and **k**. Pairwise corr. of grid cell pairs at all distances for the good (**g**) and poor (**k**) performers. **h** and **l**. Adjusted Pairwise corr. in the three zones of the good (**h**) and poor (**l**) performers. *$p \leq 0.05$, **$p \leq 0.01$, ***$p \leq 0.001$. Error bars represent mean ± sem. See Supplementary Data 1 for exact n and detailed statistical information. Source data are provided as a Source Data file.

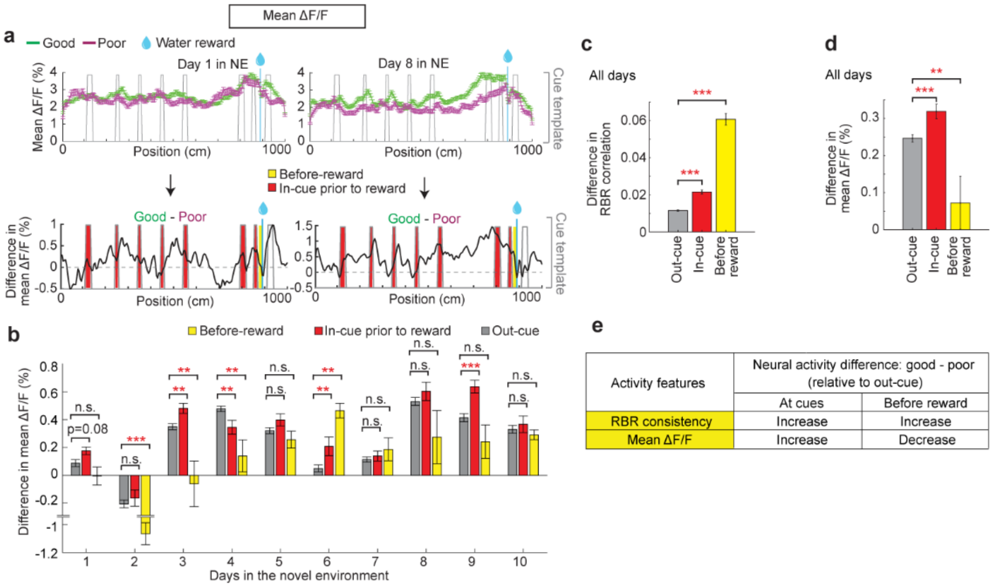

Supplementary Figure 8 Comparison of RBR activity consistency and mean ΔF/F between good and poor performers, Related to Figure 7.

a. Top: Comparison of mean activity (ΔF/F) as a function of track location between the good and poor performers on days 1 (left) and 8 (right). Bottom: The difference between mean ΔF/F in the good and poor performers. NE: Novel environment. **b**. The mean ΔF/F difference between the good and poor performers. **c** and **d**. Grouped run-by-run (RBR) activity consistency difference (**c**, data from **Figure 7b**) and mean ΔF/F difference (**d**, data from **b**) between the good and poor performers across 10 days together. **e**. Summary of neural response (RBR activity consistency and mean ΔF/F) difference between the good and poor performers at cues and before reward. $^*p \leq 0.05$, $^{**}p \leq 0.01$, $^{***}p \leq 0.001$. Error bars represent mean ± sem. See Supplementary Data 1 for exact n and detailed statistical information. Source data are provided as a Source Data file.

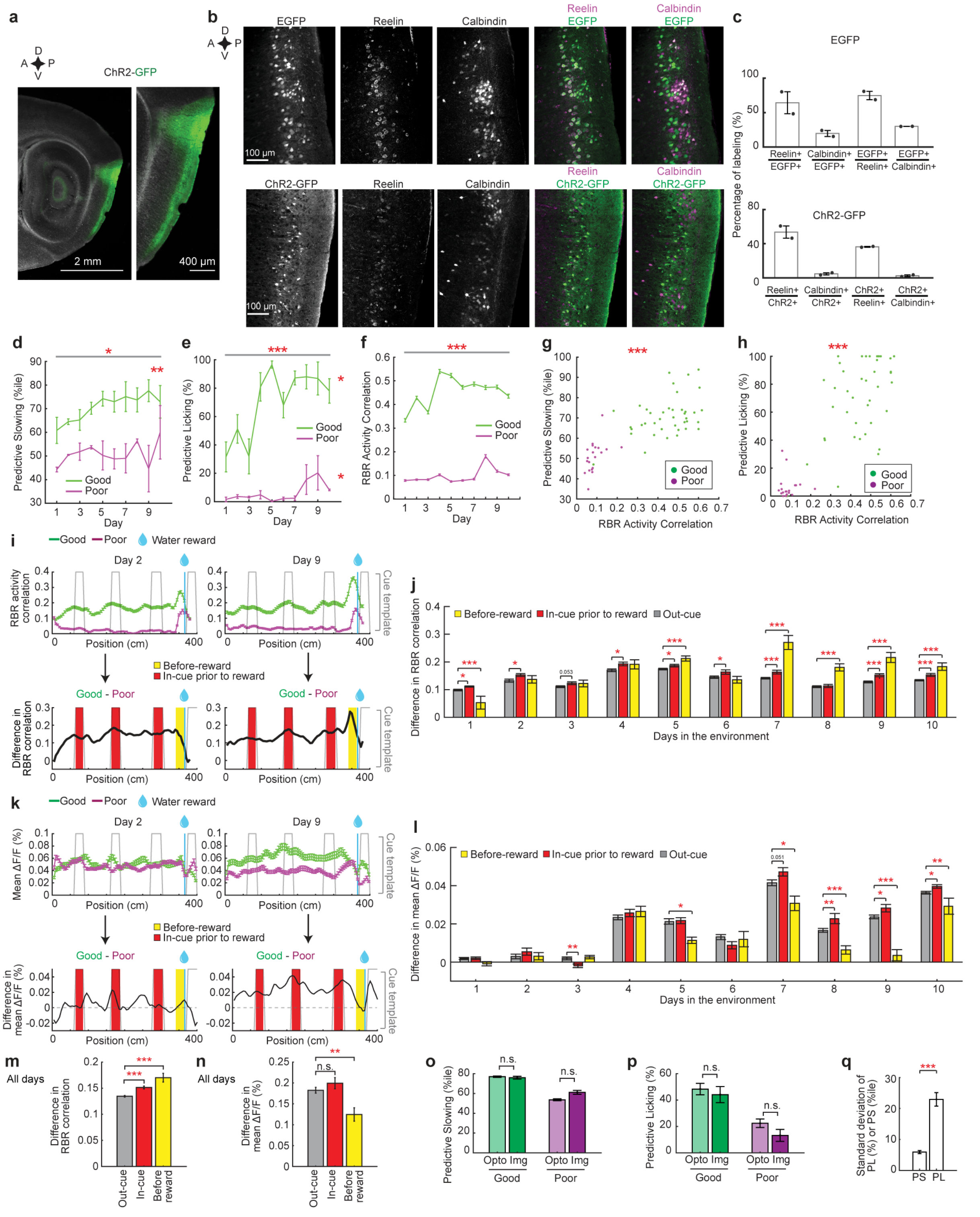

Supplementary Figure 9. Viral expression and stimulation for optogenetic experiments, Related to Figure 8

a. Epifluorescence images of sagittal MEC sections showing expression of ChR2-GFP in the superficial layers of the MEC. D: dorsal; V: ventral; A: anterior; P: posterior. **b**. Histological

staining of Reelin and Calbindin in the MEC for EGFP (upper panels) or ChR2-GFP (lower panels) injected mice. Signals from Reelin staining were adjusted for clarity using Fiji. **c**. Overlap of Reelin+ and Calbindin+ neurons with EGFP (upper panels) or ChR2-GFP (lower panels) expression. Each column represents the percentage of cells in the numerator category among cells in the denominator category. **d-f**. Predictive slowing (**d**), licking (**e**), and run-by-run (RBR) activity consistency (**f**) during the learning of the 4m track. Horizontal grey bars indicate p values for the group difference between good and poor performers. * to the right of line graph indicates significant correlation from day 1 to day 10. **g** and **h**. Correlation between RBR activity consistency and predictive slowing (**g**) or licking (**h**) of individual mice on individual days of learning. **i** and **k**. Top: Comparison of RBR spatial activity consistency (**i**) and mean activity (**k**, $\Delta F/F$) as a function of track location between the good and poor performers on days 2 (left) and 9 (right). Bottom: The difference between the good and poor performers. **j** and **l**. RBR activity correlation difference (**j**) and mean $\Delta F/F$ difference (**l**) between the good and poor performers. **m** and **n**. Grouped RBR activity consistency difference (**m**, data from **k**) and mean $\Delta F/F$ difference (**n**, data from **l**) between the good and poor performers across 10 days together. **o** and **p**. Comparison of predictive slowing (**o**) and licking (**p**) of optogenetics (Opto) and imaging (Img) mice. **q**. Standard deviation of predictive slowing (PS) and licking (PL) within the three sessions of the same mouse. *$p \leq 0.05$, **$p \leq 0.01$, ***$p \leq 0.001$. Error bars represent mean ± sem. See Supplementary Data 1 for exact n and detailed statistical information. Source data are provided as a Source Data file.

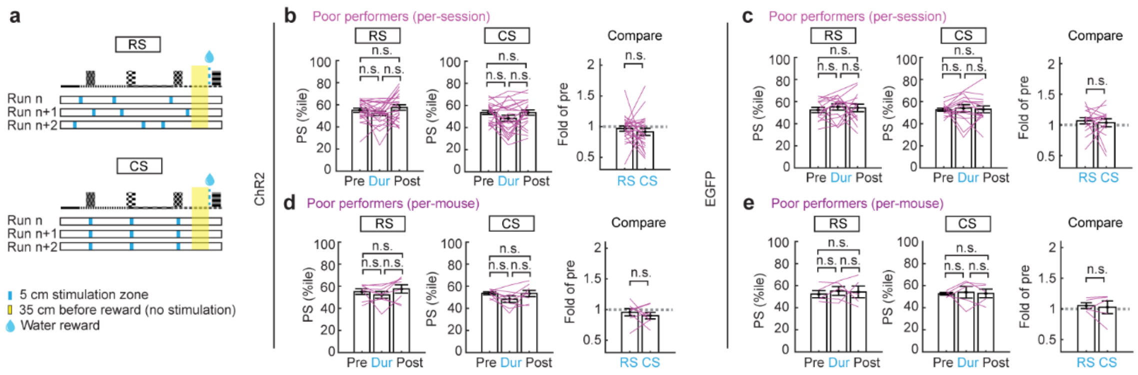

Supplementary Figure 10. Optogenetic stimulation in the poor performers, Related to Figure 8

a. Schematics showing the stimulation of three randomly located 5cm zones (RS) and consistent stimulation for 5cm at each cue preceding the reward (CS) on an RBR basis. Identical to Fig. 8f. **b-e**. Predictive slowing (PS) of ChR2 (**b** and **d**, 8 mice) and EGFP (**c** and **e**, 5 mice) poor performers pre-, during- (Dur), and post- the RS (left) and CS (middle) in **a**. Data was calculated per session (**b** and **c**, 3 sessions per mouse) and per mouse (**d** and **e**). Mean PS of 10 runs in the three periods are plotted. Right: "Fold of pre" reflects relative PS levels during RS and CS (PS levels during RS and CS divided by the PS during pre-stimulation). **b** and **c**. Since RS and CS were performed in alternation for the same mouse, the effects of adjacent RS and CS sessions were compared. *p ≤ 0.05, **p ≤ 0.01, ***p ≤ 0.001. Error bars represent mean ± sem. See Supplementary Data 1 for exact n and detailed statistical information. Source data are provided as a Source Data file.

Number of Days

	1m Training	Other VR Tracks	FE Training	Experiment Track
10m Imaging Mice	6.3+/-3.4 (3 mice)	49.0+/-19.0 (2 mice)	19.9+/-3.0	**10.0+/-0.0**
4m Imaging Mice	3.7+/-1.4	74.2+/-8.8	12.2+/-2.9	**10.0+/-0.0**
Histology Mice	NA	NA	**30.9+/-1.7**	1.0+/-0.0
Optogenetics Mice	12.0+/-1.5	NA	**34.3+/-2.8**	23.6+/-1.8

Number of Days with 10+ Laps

	1m Training	Other VR Tracks	FE Training	Experiment Track
10m Imaging Mice	4.7+/-1.8 (3 mice)	47.5+/-20.5 (2 mice)	13.6+/-1.5	**10.0+/-0.0**
4m Imaging Mice	2.8+/-1.0	68.5+/-9.1	9.8+/-1.7	**10.0+/-0.0**
Histology Mice	NA	NA	**18.8+/-1.6**	0.9+/-0.1
Optogenetics Mice	8.6+/-1.3	NA	**32.0+/-3.1**	23.5+/-1.8

Number of Laps

	1m Training	Other VR Tracks	FE Training	Experiment Track
10m Imaging Mice	301+/-79 (3 mice)	4380+/-3010 (2 mice)	425+/-29	**374+/-27**
4m Imaging Mice	159+/-56	2880+/-720	430+/-137	**336+/-26**
Histology Mice	NA	NA	**858+/-239**	51.2+/-9.1
Optogenetics Mice	494+/-66	NA	**1740+/-240**	991+/-78

Supplementary Table 1. Virtual reality training details

The number of days (top), the number of days with 10 or more laps traversed (middle), and the number of laps (bottom) that each category of experimental mice experienced in each type of environment. Of the 10m imaging mice, only 3 and 2 mice experienced 1m training and other VR tracks, respectively, so summary values include only those mice. Bold values indicate that histology and optogenetics mice received more training in FEs than imaging mice received in NEs, indicating that the former experienced truly familiar environments on experimental days. Values represent mean ± SEM. FE: Familiar Environment. NE: Novel Environment.

SUPPLEMENTARY REFERENCES

1 Kentros, C. G., Agnihotri, N. T., Streater, S., Hawkins, R. D. & Kandel, E. R. Increased attention to spatial context increases both place field stability and spatial memory. *Neuron* **42**, 283-295 (2004). https://doi.org:10.1016/s0896-6273(04)00192-8

2 Pettit, N. L., Yuan, X. C. & Harvey, C. D. Hippocampal place codes are gated by behavioral engagement. *Nat Neurosci* **25**, 561-566 (2022). https://doi.org:10.1038/s41593-022-01050-4

3 Hardcastle, K., Maheswaranathan, N., Ganguli, S. & Giocomo, L. M. A Multiplexed, Heterogeneous, and Adaptive Code for Navigation in Medial Entorhinal Cortex. *Neuron* **94**, 375-387 e377 (2017). https://doi.org:10.1016/j.neuron.2017.03.025
