# Supplementary material for: A consistent map in the medial entorhinal cortex supports spatial memory
Source: Nat Commun. 2024 Feb 17;15:1457. doi: 10.1038/s41467-024-45853-4 (PMC10874432; doi:10.1038/s41467-024-45853-4)
Supplement: Supplementary file 3 — Description of Additional Supplementary Files [file 41467_2024_45853_MOESM3_ESM.pdf]

Description of Additional Supplementary Files

File Name: Supplementary Data 1
Description: Contains exact n and p values, as well as detailed statistical information, for all Figures and Supplementary Figures.
