# Supplementary material for: A consistent map in the medial entorhinal cortex supports spatial memory
Source: Nat Commun. 2024 Feb 17;15:1457. doi: 10.1038/s41467-024-45853-4 (PMC10874432; doi:10.1038/s41467-024-45853-4)
Supplement: Supplementary file 5 — Reporting Summary [file 41467_2024_45853_MOESM5_ESM.pdf]

nature portfolio

Corresponding author(s): Yi Gu

Last updated by author(s): Jan 12, 2024

Reporting Summary

Nature Portfolio wishes to improve the reproducibility of the work that we publish. This form provides structure for consistency and transparency in reporting. For further information on Nature Portfolio policies, see our Editorial Policies and the Editorial Policy Checklist.

Statistics

For all statistical analyses, confirm that the following items are present in the figure legend, table legend, main text, or Methods section.

n/a	Confirmed	
☐	☒	The exact sample size (*n*) for each experimental group/condition, given as a discrete number and unit of measurement
☐	☒	A statement on whether measurements were taken from distinct samples or whether the same sample was measured repeatedly
☐	☒	The statistical test(s) used AND whether they are one- or two-sided<br>*Only common tests should be described solely by name; describe more complex techniques in the Methods section.*
☐	☒	A description of all covariates tested
☐	☒	A description of any assumptions or corrections, such as tests of normality and adjustment for multiple comparisons
☐	☒	A full description of the statistical parameters including central tendency (e.g. means) or other basic estimates (e.g. regression coefficient) AND variation (e.g. standard deviation) or associated estimates of uncertainty (e.g. confidence intervals)
☐	☒	For null hypothesis testing, the test statistic (e.g. *F*, *t*, *r*) with confidence intervals, effect sizes, degrees of freedom and *P* value noted<br>*Give P values as exact values whenever suitable.*
☒	☐	For Bayesian analysis, information on the choice of priors and Markov chain Monte Carlo settings
☒	☐	For hierarchical and complex designs, identification of the appropriate level for tests and full reporting of outcomes
☐	☒	Estimates of effect sizes (e.g. Cohen's *d*, Pearson's *r*), indicating how they were calculated

Our web collection on statistics for biologists contains articles on many of the points above.

Software and code

Policy information about availability of computer code

Data collection	Two-photon imaging was performed using Prairie View (Bruker, version 5.5). Mouse behavior was recorded using ViRMEn (Princeton, version 2016-02-12) and Prairie View. Electrophysiology recordings were performed using Open Ephys GUI (Open Ephys, version 0.5.5.2).
Data analysis	Image processing was performed using previously published MATLAB (Mathworks, version R2015aSP1) codes as cited in the methods section. Data analysis was performed using ImageJ (Fiji, version 1.53q), MATLAB (Mathworks, versions R2015aSP1 and 2020a), and GraphPad Prism (version 9.3.1). Custom MATLAB scripts used for data analysis are available on Github (https://github.com/GuLab-NIH/MaloneNatComm2024).

For manuscripts utilizing custom algorithms or software that are central to the research but not yet described in published literature, software must be made available to editors and reviewers. We strongly encourage code deposition in a community repository (e.g. GitHub). See the Nature Portfolio guidelines for submitting code & software for further information.

Data

Policy information about availability of data

All manuscripts must include a data availability statement. This statement should provide the following information, where applicable:

- Accession codes, unique identifiers, or web links for publicly available datasets
- A description of any restrictions on data availability
- For clinical datasets or third party data, please ensure that the statement adheres to our policy

Raw data are extremely large and not feasible for upload to an online repository but are available upon request to yi.gu@nih.gov. Processed source data for all figures and associated statistical analysis are provided with the paper. Source data are provided with this paper.

Research involving human participants, their data, or biological material

Policy information about studies with human participants or human data. See also policy information about sex, gender (identity/presentation), and sexual orientation and race, ethnicity and racism.

Reporting on sex and gender	Human research participants were not used.
Reporting on race, ethnicity, or other socially relevant groupings	Human research participants were not used.
Population characteristics	Human research participants were not used.
Recruitment	Human research participants were not used.
Ethics oversight	Human research participants were not used.

Note that full information on the approval of the study protocol must also be provided in the manuscript.

Field-specific reporting

Please select the one below that is the best fit for your research. If you are not sure, read the appropriate sections before making your selection.

☒ Life sciences ☐ Behavioural & social sciences ☐ Ecological, evolutionary & environmental sciences

For a reference copy of the document with all sections, see nature.com/documents/nr-reporting-summary-flat.pdf

Life sciences study design

All studies must disclose on these points even when the disclosure is negative.

Sample size	No statistical methods were used to predetermine sample sizes. Sample sizes in terms of mice and neurons are similar to other contemporary studies in the field (For example, see Pettit et. al. Nature 2022, Kinkhabwala et. al. eLife 2020, Robinson et. al. Cell 2020, Gu et. al. Cell 2018, Danielson et. al. Neuron 2016).
Data exclusions	One mouse that did not learn to run in virtual reality after viral injection was excluded from the optogenetics experiments.
Replication	All experiments were replicated at least once, and similar results were obtained.
Randomization	Good and poor performing mouse groups were determined based on behavioral performance as described in the text. Mice in optogenetics experiments experienced both consistent and random stimulation. As such, mice in the imaging and optogentics experiments experienced the same experimental conditions, making randomization irrelevant. Animals were randomly allocated to the different experimental conditions (FE versus NE) for the histology experiments.
Blinding	All data collection was blind as to mouse performer group, as performance was analyzed after the conclusion of data collection. All data analysis was blinded, as it was performed using automated code.

Reporting for specific materials, systems and methods

We require information from authors about some types of materials, experimental systems and methods used in many studies. Here, indicate whether each material, system or method listed is relevant to your study. If you are not sure if a list item applies to your research, read the appropriate section before selecting a response.

Materials & experimental systems

n/a	Involved in the study
☐	☒ Antibodies
☒	☐ Eukaryotic cell lines
☒	☐ Palaeontology and archaeology
☐	☒ Animals and other organisms
☒	☐ Clinical data
☒	☐ Dual use research of concern
☒	☐ Plants

Methods

n/a	Involved in the study
☒	☐ ChIP-seq
☒	☐ Flow cytometry
☒	☐ MRI-based neuroimaging

Antibodies

Antibodies used

Primary antibodies included: Rabbit anti-c-Fos (9F6) (Cell Signaling Technology: 2250S, Lot # 11); Mouse IgG1 anti-Reelin (G10) (Abcam: AB78540, Lot # gr3315810-1); Mouse IgG2a anti-Calbindin (AF2E5) (Abcam: AB75524, Lot # gr3380191-1); Mouse IgG2a anti-GAD67 (1G10.2) (MilliporeSigma: MAB5406, Lot # 3445024)
Secondary antibodies included: Alexa 488 anti-rabbit (ThermoFisher Scientific: A32731, Lot # VC297825);
Alexa 568 anti-mouse IgG1 (ThermoFisher Scientific: A-21124, Lot # 2129006); Alexa 568 anti-mouse IgG2a (ThermoFisher Scientific: A-21134, Lot # 2185075); Alexa 647 anti-mouse IgG1 (ThermoFisher Scientific: A-21241, Lot # 2349091)

Validation

Rabbit anti-c-Fos (Cell Signaling Technology, 2250S) has been validated by the manufacturer. As stated by Cell Signaling Technology, the antibody has been validated in H, M, R, for WB, W-S, IF-F, IF-IC, FC-FP, and ChIP with at least 803 citations. RRID:AB_2247211. Mouse anti-Reelin (1:1000, Abcam, AB78540) has been validated by the manufacturer. As stated by Abcam, the antibody has been validated in H, M, R, for WB and IHC with at least 30 citations. RRID:AB_1603148. Mouse anti-Calbindin (1:3000, Abcam, AB75524) has been validated by the manufacturer. As stated by Abcam, the antibody has been validated in H, M, R, for WB and IHC with at least 8 citations. RRID:AB_1310017. Mouse anti-GAD67 (1:2000, Millipore Sigma, MAB5406) has been validated by the manufacturer. As stated by Millipore Sigma, the antibody has been validated in H, M, R, for ELISA, IHC-FoFr, Flow Cyt, ChIP, ICC/IF, and WB with at least 709 citations. RRID:AB_2278725.

Animals and other research organisms

Policy information about studies involving animals; ARRIVE guidelines recommended for reporting animal research, and Sex and Gender in Research

Laboratory animals

GP5.3 (C57BL/6J-Tg(Thy1-GCaMP6f)GP5.3Dkim/J, JAX stock #028280) mice : 4-8 months old
C57BL/6J: - months old
TRAP2 mice (Fostm2.1(icre/ERT2)Luo/J, Jackson stock #030323) crossed with CAG-Sun1/sfGFP (B6;129-Gt(ROSA)26Sortm5(CAGSun1/sfGFP)Nat/J, Jackson stock # 021039) or Ai14 (B6;129S6-Gt(ROSA)26Sortm14(CAG-tdTomato)Hze/J, Jackson stock #007908) to generate TRAP2+/-;CAG-Sun1/sfGFP+/- or TRAP2+/-;Ai14+/-: 2.5-4 months old.
Details are included in the methods. Mice were maintained on a reverse 12-hr on/off light schedule with all experiments being performed in the light off period. Animals were house at a temperature of 70–74°F and 40–65% humidity.

Wild animals

No wild animals were used.

Reporting on sex

Both male and female mice were used for all experiments based on availability at the time of experiment and to ensure roughly equal numbers of each sex. Sex differences were not found in predictive slowing and predictive licking (Extended Data Figure 1m, n), so additional analyses did not distinguish by sex.
GP5.3 mice (two-photon imaging): 8 males and 7 females
C57BL/6J mice (optogenetics experiments): males and females
TRAP2+/-;CAG-Sun1/sfGFP+/- (histology experiments): 4 males
TRAP2+/-;Ai14+/- (histology experiments): 6 males and 10 females

Field-collected samples

Study did not involve field-collected samples.

Ethics oversight

All animal procedures were performed in accordance with animal protocol 1524 approved by the Institutional Animal Care and Use Committee (IACUC) at NIH/NINDS.

Note that full information on the approval of the study protocol must also be provided in the manuscript.

Plants

Seed stocks	*Report on the source of all seed stocks or other plant material used. If applicable, state the seed stock centre and catalogue number. If plant specimens were collected from the field, describe the collection location, date and sampling procedures.*
Novel plant genotypes	*Describe the methods by which all novel plant genotypes were produced. This includes those generated by transgenic approaches, gene editing, chemical/radiation-based mutagenesis and hybridization. For transgenic lines, describe the transformation method, the number of independent lines analyzed and the generation upon which experiments were performed. For gene-edited lines, describe the editor used, the endogenous sequence targeted for editing, the targeting guide RNA sequence (if applicable) and how the editor was applied.*
Authentication	*Describe any authentication procedures for each seed stock used or novel genotype generated. Describe any experiments used to assess the effect of a mutation and, where applicable, how potential secondary effects (e.g. second site T-DNA insertions, mosiacism, off-target gene editing) were examined.*
